# Risk of sudden sensorineural hearing loss in patients with dysrhythmia: A nationwide population-based cohort study

**DOI:** 10.1371/journal.pone.0218964

**Published:** 2019-06-26

**Authors:** Chih-Wei Luan, Jung-Jung Chang, Cheng-Ming Hsu, Ming-Shao Tsai, Geng-He Chang, Ethan I. Huang, Ku-Hao Fang, Meng-Hung Lin, Chia-Yen Liu, Yao-Hsu Yang, Yao-Te Tsai

**Affiliations:** 1 Department of Otorhinolaryngology-Head and Neck Surgery, Chang Gung Memorial Hospital, Chiayi, Taiwan; 2 Division of Cardiology, Chang Gung Memorial Hospital, Chiayi, Taiwan; 3 Department of Otorhinolaryngology-Head and Neck Surgery, Chang Gung Memorial Hospital, Linkou, Taiwan; 4 Health Information and Epidemiology Laboratory, Chang Gung Memorial Hospital, Chiayi, Taiwan; 5 Department of Traditional Chinese Medicine, Chang Gung Memorial Hospital, Chiayi, Taiwan; Center for Healthy Start Initiative, NIGERIA

## Abstract

**Objective:**

Whether dysrhythmia is a risk factor of sudden sensorineural hearing loss (SSNHL) remains unclear. In this study, we aimed to investigate the risk of developing SSNHL among patients with dysrhythmia in different age and gender groups by using population-based data in Taiwan.

**Methods:**

We conducted a matched cohort study by analyzing data between January 2000 and December 2013 obtained from the Taiwan National Health Insurance Research Database. 41,842 newly diagnosed dysrhythmia patients and 83,684 comparison subjects without dysrhythmia were selected from claims. The incidence of sudden sensorineural hearing loss at the end of 2013 was determined in both groups. Univariate and multivariate logistic regression analyses were used to investigate the risk of SSNHL among patients with dysrhythmia.

**Results:**

The incidence of SSNHL was 1.30-fold higher in the dysrhythmia group compared with the control group (53.2 versus 40.9 per 100,000 person-years), and using Cox proportional hazard regressions, the adjusted hazard ratio (HR) was 1.40 (95% confidence interval [CI], 1.15–1.70). Gender-stratified analysis revealed a significantly higher risk of SSNHL in patients with dysrhythmia than in those without dysrhythmia for both men and women (HR = 1.34, 95% CI = 1.02–1.76, *P* = 0.039, HR = 1.35, 95% CI = 1.02–1.78, *P* = 0.035, respectively). Age-stratified analysis revealed remarkable associations between dysrhythmia and SSNHL among those aged less than 40 years and more than 65 years (HR = 2.18, 95% CI = 1.03–4.64, *P* = 0.043 and HR = 1.54, 95% CI = 1.14–2.09, *P* = 0.006, respectively).

**Conclusions:**

Our findings support dysrhythmia as an independent risk factor for SSNHL. Based on the study results, clinicians managing patients with dysrhythmia should be aware of the increased risk of developing SSNHL, especially among patients aged <40 and >65 years, and counsel patients to seek medical advice immediately if they experience any acute change in their hearing ability.

## Introduction

Sudden sensorineural hearing loss (SSNHL) is an otologic emergency and defined as an acute loss of 30 dB or more in at least three contiguous audiometric frequencies over a period of less than 3 days [1]. The incidence of SSNHL was reported to range from 5 to 20 per 100,000 individuals and increase with age [2]. Predominant occurrence of left-side SSNHL has been observed, and vertebrobasilar curvature may explain the laterality of this disease [3,4]. Previous studies have reported increased risk of developing SSNHL among patients with diabetes mellitus, systemic lupus erythematosus, chronic kidney disease, chronic otitis media, osteoporosis, psoriasis, iron deficiency anemia, and human immunodeficiency virus [5–12]. Despite the great advances in otology over the past decades, the causes of SSNHL remain controversial, and SSNHL seems to be a multifactorial disease. Chau et al. conducted a systematic review of 23 articles and determined that the likely etiologies for SSNHL were 71.0% idiopathic, 12.8% from infection, 4.7% otologic, 4.2% from trauma, 2.8% from vascular or hematologic disorders, 2.3% from neoplasm, and 2.2% from other causes [13]. Four major pathogenetic mechanisms have been proposed to explain SSNHL: vascular compromise, viral infection, rupture of the intracochlear membrane, and autoimmune inner ear disease. Among these, impairment of cochlear perfusion appears to be the most crucial event [14,15].

Dysrhythmia is associated with higher risks of several severe adverse effects on various body systems, such as higher risk of hypertension, stroke, myocardial infarction, and increased mortality from both congestive heart failure and stroke [16–19]. Dysrhythmia not only leads to systemic hemodynamic instability but also is associated with increased risks of thromboembolism and atherosclerosis, both of which have been reported to be related to SSNHL and chronic hearing impairment [20–26]. Previous studies have also found that patients with SSNHL exhibit autonomic alterations presenting as dysregulation in heart rhythm and blood pressure [27–29].

Given a possible link between dysrhythmia and SSNHL, it is rational to hypothesize an interrelationship between the clinical profiles of SSNHL and patients with dysrhythmia. The theoretical expectation, however, has yet to be fully elucidated, and a lack of relevant evidence precludes the formation of clear recommendations regarding the clinical practice. Deeper comprehension of SSNHL risk in patients with dysrhythmia is needed; therefore, we conducted the first study investigating the association between dysrhythmia and SSNHL using a large-scale population-based cohort in Taiwan to test our hypothesis.

## Material and methods

### Ethics statement

The study protocol was reviewed and approved by the Institutional Review Board (IRB) of Chang Gung Memorial Hospital (approval no. 201801568B1).

### Data source

The Taiwanese government implemented a compulsory National Health Insurance (NHI) program in March 1995, which is a nationwide health care system and provides medical services for the country’s 23.5 million residents. It covers over 99% of the population in Taiwan and records clinical diagnoses based on the International Classification of Diseases, Ninth Revision, Clinical Modification (ICD-9-CM) codes [30,31]. The high-coverage rate of the NHI enables studies based on the NHI Research Database (NHIRD) to be nationwide and population based. The NHIRD contains comprehensive information on prescription details, clinic visits, surgical procedures, and diagnostic codes. The personal information of all patients included in the NHIRD is encrypted to ensure their privacy.

This study retrieved data from the Longitudinal Health Insurance Database 2000 (LHID 2000), which is a representative database of the NHIRD. The LHID 2000 includes all medical claims (from 1996 to 2013) of one million individuals randomly selected from the 2000 Registry of Beneficiaries of the NHIRD using a systematic sampling method, representing approximately 5% of people in Taiwan. According to the Taiwan National Health Research Institutes reports, no statistically significant differences exist in age, gender, or health care costs between the sample group and all enrollees in the LHID 2000 [32].

This study was reviewed and approved by the institutional review board of Chang Gung Memorial Hospital.

### Study design and participants

**Fig 1:**
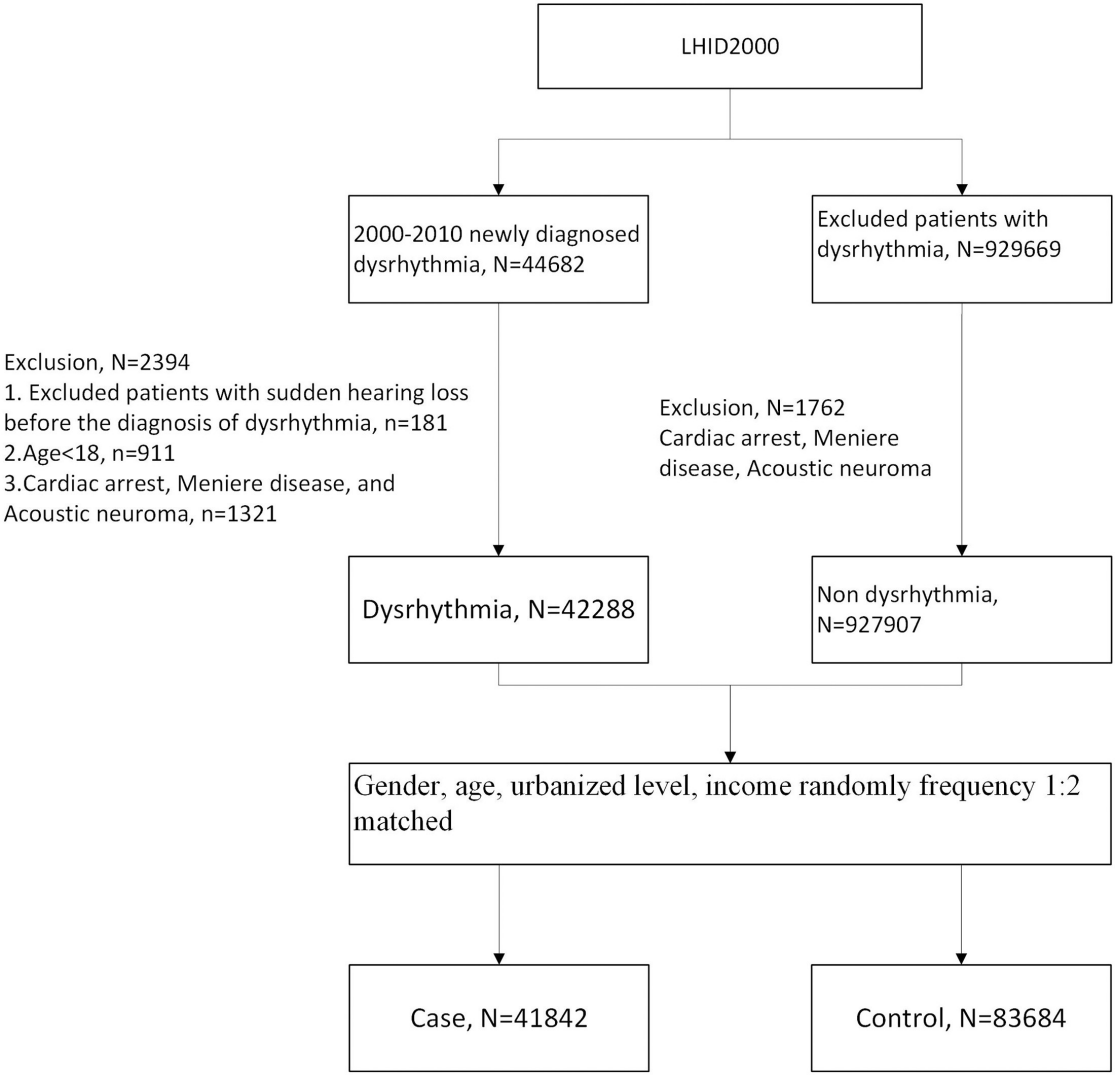
Flow diagram of the study. LHID, Longitudinal Health Insurance Database.

The flowchart of the patient enrollment for the study group is showned in the Fig 1. The study group was identified from the LHID 2000 and included patients aged ≥18 years who were newly diagnosed with dysrhythmia (ICD-9-CM codes 427.xx and 426.xx) between January 1, 2000, and December 31, 2010 [33,34].

We only included patients with dysrhythmia if the ICD-9-CM was coded by a cardiologist in either an inpatient setting or in three or more ambulatory care claims. These strict criteria enhanced the accuracy of the diagnosis of dysrhythmia. The date of enrollment was defined as the date of the initial diagnosis of dysrhythmia. The comparison cohort included patients without dysrhythmia who were matched in a 1:2 ratio by age, gender, urbanization level of the patient’s residence, and monthly income, and was randomly selected from the same data sets. For the comparison group, the start of the follow-up was defined as the date of the first visit to a medical facility in the year of enrollment. In both groups, patients diagnosed with cardiac arrest (ICD-9-CM codes 427.5), Meniere disease, or acoustic neuroma before enrollment or sudden hearing loss before the diagnosis of dysrhythmia were excluded from the study.

### Outcome and covariate measurements

Patients were followed up to the end of the study period (December 31, 2013) or death. Death was defined as until the end of the study period, withdrawal from the NHI program, or death [31].

The primary outcome was SSNHL (ICD-9-CM code 388.2). Patients’ sociodemographic data, including age, gender, urbanization level of residence, and monthly income, were obtained from initial enrollment data in the NHIRD. Data regarding the following comorbidities associated with SSNHL were retrieved from ambulatory and inpatient claims data: coronary artery disease (ICD-9-CM codes 410–414, A270, A279), diabetes mellitus (ICD-9-CM code 250.xx and A-code A-181), hyperlipidemia (ICD-9-CM codes 272.0–272.4), otitis media (ICD-9-CM codes 381.1–381.3; 382.1–382.3), hypertension (ICD-9-CM codes 401–405), chronic kidney disease (ICD-9-CM codes 582–588), systemic lupus erythematosus (ICD-9-CM codes 710.0), and stroke (ICD-9-CM codes 430–438). We included these comorbidities if they occurred either in the inpatient setting or were found in three or more ambulatory care claims. Each comorbid condition was analyzed as a binary variable.

### Statistical analysis

The descriptive statistics and continuous variables were analyzed using the Pearson chi-squared test and independent Student’s t test, respectively, to compare the sociodemographic characteristics and comorbidities between the dysrhythmia group and comparison group.

The incidence rate was calculated as the number of SSNHL cases diagnosed in the follow-up period divided by the total person–years for each group by gender, age, and number of follow-up years. The risk of developing SSNHL was compared between the dysrhythmia group and the control group by estimating the incidence rate ratio through Poisson regression.

Kaplan–Meier methods were used to calculate the cumulative incidence rates of SSNHL between the two groups, and the logrank test was used to analyze the differences in the incidence curves between the two groups. The adjusted hazard ratio (HR) for SSNHL occurrence was estimated using Cox proportional hazard models. All statistical analyses were performed using SAS version 9.4 (SAS Institute, Cary, NC, USA), and the statistical significance was set at two-sided *P* < 0.05.

## Results

The study cohorts comprised 41,842 patients with dysrhythmia and 83,684 comparison patients without dysrhythmia. After patients were matched by gender, age, and socioeconomic status, the dysrhythmia group appeared more prone to comorbid coronary artery disease, diabetes mellitus, hyperlipidemia, otitis media, hypertension, chronic kidney disease, systemic lupus erythematosus, and stroke than the comparison group (Table 1). In this study setting, the mean (standard deviation) observation duration was 7.8 (3.7) years for the dysrhythmia group and 8.3 (3.4) years for the control group.

**Table 1:** Demographic characteristics and comorbidities of dysrhythmia patients and patients of the control group.

Variable	Dysrhythmia (N = 41842)	Non- Dysrhythmia (N = 83684)	*p*-value
Gender			
Men	20098 (48.0)	40196 (48.0)	1.000
Women	21744 (52.0)	43488 (52.0)	
Age (years)			
<40	5245 (12.5)	10490 (12.5)	1.000
40–65	17114 (40.9)	34228 (40.9)	
≧65	19483 (46.6)	38966 (46.6)	
SSNHL			
Yes	174 (0.4)	284 (0.4)	0.034
No	41668 (99.6)	83400 (99.7)	
Baseline comorbidities			
CAD	10462 (25.0)	7543 (9.0)	<0.0001
DM	5972 (14.3)	8138 (9.7)	<0.0001
Hyperlipidemia	8491 (20.3)	11424 (13.7)	<0.0001
Otitis media	325 (0.8)	463 (0.6)	<0.0001
Hypertension	8192 (19.6)	14139 (16.9)	<0.0001
CKD	5021 (12.0)	5673 (6.8)	<0.0001
SLE	72 (0.2)	87 (0.1)	0.0014
Stroke	7008 (16.8)	7778 (9.3)	<0.0001
Urbanized level			
1 (City)	12009 (28.7)	24018 (28.7)	1.000
2	17845 (42.7)	35690 (42.7)	
3	7722 (18.5)	15444 (18.5)	
4 (Villages)	4266 (10.2)	8532 (10.2)	
Monthly income			
NT < 15,840	16236 (38.8)	32472 (38.8)	1.000
NT 15,841~25,000	18719 (44.7)	37438 (44.7)	
NT > 25,001	6887 (16.5)	13774 (16.5)	

*Note*. Numbers in parentheses represent percentages.

SSNHL = Sudden sensorineural hearing loss; CAD = Coronary artery disease;

DM = Diabetes mellitus; CKD = chronic kidney disease; SLE = systemic lupus erythematosus

NT = New Taiwan dollar

**Fig 2:**
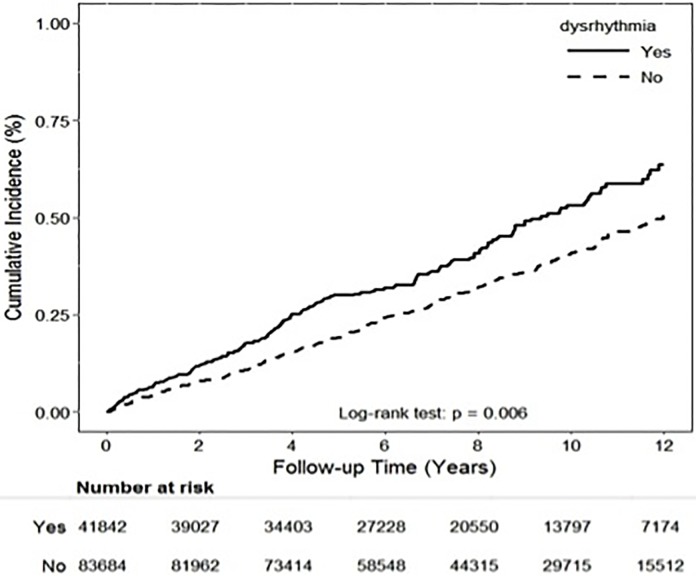
SSNHL incidence in the dysrhythmia group and the non- dysrhythmia control group in Taiwan (2000–2013).

Using the Kaplan–Meier approach, the cumulative incidence of SSNHL was found to be significantly higher in patients with dysrhythmia than in those without dysrhythmia (*P* = 0.006; Fig 2). The incidence of SSNHL in the dysrhythmia group was 1.30-fold that in the comparison group (53.2 vs. 40.9 per 100,000 person–years; Table 2). A stratified analysis of the duration of follow-up showed a significantly higher incidence of developing SSNHL in the dysrhythmia group compared with the control group when stratified by 1–5 follow-up years *(P* = 0.002; Table 2).

**Table 2:** Risk of SSNHL for dysrhythmia group and control group.

	Dysrhythmia				Controls				IRR(95% CI)	p-value
	n	SSNHL	Person-yeras	Rate†	n	SSNHL	Person-years	Rate†		
Overall	41841	174	327094.2	53.2	83684	284	694662.0	40.9	1.30(1.08–1.57)	0.006
Years of follow-up										
<1	41842	27	40552.4	66.6	83684	34	83237.2	40.8	1.63(0.98–2.70)	0.058
1–5	39904	86	146876.4	58.6	82806	117	310640.1	37.7	1.55(1.18–2.05)	0.002
>5	30707	61	131637.7	46.3	65800	133	300784.6	44.2	1.05(0.77–1.42)	0.762

^†^Rate: per 100,000 person-years; IRR = Incidence rate ratio; incidence rate ratio was compared using Poisson regression.

Table 3 presents the results of multivariate analysis comparing the risk of SSNHL between the dysrhythmia group and control group. Patients with dysrhythmia showed a significantly higher risk of SSNHL (adjusted HR [95% confidence interval, CI]: 1.40 [1.15–1.70] for the full model and 1.30 [1.07–1.57] for the main model) than those without dysrhythmia. The main model was adjusted for gender, age group, urbanization level, and monthly income, whereas the full model was adjusted for gender, age group, urbanization level, income, and all comorbidities. Table 3 also demonstrated that the effect was similar in the sensitivity analyses of other potential confounding variables, including coronary artery disease, diabetes mellitus, hyperlipidemia, otitis media, hypertension, chronic kidney disease, systemic lupus erythematosus, and stroke. The HRs remained stable between 1.29 and 1.34 (all *P* value < 0.05).

**Table 3:** Risk of sudden sensorineural hearing loss was compared by multivariable Cox regression model.

Variable	Adjusted HR		95% CI	*p*-value
Main model*	1.30	1.07	1.57	0.007
Full model	1.40	1.15	1.70	0.001
Additional covariates†				
Main model + CAD	1.32	1.09	1.60	0.005
Main model + DM	1.32	1.10	1.60	0.004
Main model + Hyperlipidemia	1.33	1.10	1.61	0.004
Main model + Otitis media	1.29	1.07	1.56	0.008
Main model + Hypertension	1.30	1.08	1.58	0.006
Main model + CKD	1.33	1.10	1.61	0.003
Main model + SLE	1.30	1.07	1.57	0.007
Main model + Stroke	1.34	1.11	1.62	0.002
Subgroup effects ‡				
Gender				
Men	1.34	1.02	1.76	0.0039
Women	1.35	1.02	1.78	0.0035
Age				
<40	2.18	1.03	4.64	0.043
40–65	1.15	0.88	1.51	0.315
≧65	1.54	1.14	2.09	0.006

Abbreviations: HR, hazard ratio; CI, confidence incidence; CAD, coronary artery disease;

DM, diabetes mellitus; CKD = chronic kidney disease; SLE = systemic lupus erythematosus

*Main model was adjusted by gender, age, urbanization level and income.

Full model was adjusted by gender, age, urbanization level, income and comorbidities.

^†^The models were adjusted for comorbidities in the main model as well as each additional listed comorbidity.

^‡^Subgroup effects were adjusted by gender, age, urbanization, income and comorbidities.

Gender-stratified analysis revealed a significantly higher risk of SSNHL in patients with dysrhythmia than in those without dysrhythmia for both men and women (HR = 1.34, 95% CI = 1.02–1.76, *P* = 0.039, HR = 1.35, 95% CI = 1.02–1.78, *P* = 0.035, respectively). Age-stratified analysis revealed remarkable associations between dysrhythmia and SSNHL among those aged less than 40 years and more than 65 years (HR = 2.18, 95% CI = 1.03–4.64, *P* = 0.043 and HR = 1.54, 95% CI = 1.14–2.09, *P* = 0.006, respectively).

## Discussion

To the best of our knowledge, this is the first study to investigate the risk of SSNHL in patients with dysrhythmia. The results showed that the risk of SSNHL was significantly higher in patients with dysrhythmia than in those without dysrhythmia, independent of confounding variables including coronary artery disease, diabetes mellitus, hyperlipidemia, and otitis media, hypertension, chronic kidney disease, systemic lupus erythematosus, and stroke. The adjusted HR for SSNHL was 1.40 for the dysrhythmia group in comparison with the non-dysrhythmia group after adjustment for confounding variables. It would be difficult for a single medical institute to collect samples of adequate size with sufficient follow-up time to investigate the long-term incidence of SSNHL after diagnosis of dysrhythmia. Using the nationwide population-based database in Taiwan, we were able to trace nearly all cases of dysrhythmia and SSNHL with minimized selection bias because all health care services are covered by the NHI program. Based on the power of a large sample size, our study also found strong evidence for the higher occurrence of SSNHL in patients with dysrhythmia. The logrank test indicated a significantly higher incidence rate of SSNHL in patients with dysrhythmia than in the comparison cohort. In our study, the mean observation duration was 7.8 ± 3.7 years in the dysrhythmia group and 8.3 ± 3.4 years in the control group. This duration was sufficient to observe the trends and changes in the risk of SSNHL between these two groups.

To define the sequence of events, we excluded patients who had been diagnosed with SSNHL before dysrhythmia. To reduce the effects of potential confounders, we used HRs (after adjustment for coronary artery disease, diabetes mellitus, hyperlipidemia, otitis media, hypertension, chronic kidney disease, systemic lupus erythematosus, and stroke) to compare the outcomes of the study and control groups. Moreover, sensitivity and subgroup analyses were performed to confirm the constant effect of dysrhythmia in all subgroups. Accordingly, our study results provide robust evidence supporting dysrhythmia as an independent risk factor for SSNHL.

The link between inner ear disorders and hemodynamic instability has been widely outlined and observed clinically in both children and adult patients [35–38]. Our study elucidated that the risk for SSNHL is higher in patients with dysrhythmia than in the general population, even after adjustment of confounders, suggesting the involvement of other factors intrinsically linked to dysrhythmia and independent of the development of middle ear disease (e.g., otitis media) [11]. Although the pathophysiological mechanism underlying the association between dysrhythmia and SSNHL remains uncertain, impaired blood perfusion to the inner ear, which is particularly sensitive to circulatory alterations because of a lack of sufficient collateral circulation, may contribute to this observation [39]. Previous studies have suggested that thromboembolism formation, atherosclerosis, and consequences of ischemia resulting from dysrhythmia may change the supply of blood to the inner ear and may carry increased risk of hearing impairment and SSNHL [3,21,22,40–43]. The effectiveness of anticoagulant treatment in low-sloping SSNHL further supports the importance of thromboembolism in the pathogenesis of sudden deafness [44]. Another possible mechanism for the involvement of dysrhythmia with SSNHL is through its association with systemic inflammation, which has been found to be related to SSNHL. Studies suggest that dysrhythmia is associated with chronic inflammation, which plays a critical role in the pathogenesis and prognosis of SSNHL [45–48]. There is also evidence that the oxidative stress resulting from the atherosclerosis is related to SSNHL [49]. The neutrophil to lymphocyte ratio, an inflammatory marker of systemic inflammation, has been reported to be significantly higher in patients with dysrhythmia than in controls and is a novel diagnostic and prognostic indicator of SSNHL [50–53]. Other indicators of inflammation, such as platelet to lymphocyte ratio, C-reactive protein, and procalcitonin, have also been reported to be useful prognostic markers for SSNHL [47, 54–56]. All the aforementioned studies have supported the associations among dysrhythmia, systemic inflammation, and SSNHL.

The gender-stratified analysis showed a significantly higher risk of SSNHL in patients with dysrhythmia than in those without dysrhythmia for both men and women. Several studies have suggested that the incidence of SSNHL was higher in men than in women [57,58]. However, when gender was used as a factor that resulted in SSNHL, we found both genders were significant for patients with dysrhythmia when compared with patients without dysrhythmia. Our study also demonstrated that the contribution of dysrhythmia to the risk of SSNHL was higher among those aged less than 40 years and older than 65 years. Patients aged less than 40 years are generally accepted to be relatively healthy with few comorbidities, and the effect of dysrhythmia on the risk of SSNHL may be more apparent in this age group. Pirodda et al. analyzed 36 patients with SSNHL aged 40 years or younger and suggested that cochlear damage in these young patients may be caused by a perfusion deficit caused by cardiovascular instability; our study supports this argument [38]. By contrast, in patients with dysrhythmia aged older than 65 years, aging and increases in cardiovascular comorbidity may worsen the perfusion deficit to the inner ear and, therefore, strengthen the contribution of dysrhythmia to the risk of SSNHL [59, 60].

The present study, with the strength of its large sample size, indicates an elevated risk among patients with dysrhythmia of developing SSNHL. The large, population-based data set from Taiwan allowed us to examine the risk factors for developing SSNHL with a low chance of selection bias. However, our study has some limitations. First, the diagnosis of SSNHL was based on secondary claims data rather than audiology examinations. Detailed audiometry data were not available; therefore, we could not correlate dysrhythmia with the severity of SSNHL. Second, more frequent hospital visits by patients with dysrhythmia than the general population may result in potential surveillance bias because they would have easier access to audiology examinations. Although this would overestimate the risk of SSNHL related to dysrhythmia, audiology examination is not a routine procedure during the therapeutic monitoring of dysrhythmia [61]. Third, some degrees of bias may occur because data regarding some suspected contributing risk factors of SSNHL were not available from the insurance data, such as personal history of alcohol and cigarette consumption and previous occupational or recreational noise exposure [62]. This resulted in a confounding covariate for which it is difficult to adjust. Fourth, this population-based study has its inherent limitation in exploring the underlying mechanism by which dysrhythmia was related to SSNHL. Although we proposed possible pathogenesis, such as hemodynamic change, to correlate dysrhythmia and SSNHL, not all kinds of dysrhythmia will lead to the systemic hemodynamic instability. Besides, correlation of the effect of anti-arrhythmic agents on the following occurrence of SSNHL is of interest. However, there are a variety of treatments of different types of dysrhythmia, including catheter ablation, anti-arrhythmic agents, as well as conservative treatment, and such investigations are beyond the scope of this study. Finally, our study was retrospective. Prospective clinical trials are mandatory to elucidate the causal relationship between dysrhythmia and SSNHL and to determine whether treatment of dysrhythmia affects the onset rate of SSNHL. Although the results of our study reached statistical significance and further explored the pathogenesis of SSNHL, we remind our readers to consider these limitations when interpreting the results of this study.

In conclusion, this study was the first to elucidate the association between dysrhythmia and SSNHL using a population-based database in Taiwan. Our findings support dysrhythmia as an independent risk factor for SSNHL. Based on the study results, clinicians managing patients with dysrhythmia should be aware of the increased risk of developing SSNHL, especially among patients aged <40 and >65 years, and counsel patients to seek medical advice immediately if they experience any acute change in their hearing ability.
